# Correlates of smoking initiation among young adults in Ukraine: a cross-sectional study

**DOI:** 10.1186/1471-2458-7-106

**Published:** 2007-06-11

**Authors:** Tatiana I Andreeva, Konstantin S Krasovsky, Daria S Semenova

**Affiliations:** 1 School of Public Health, National University of Kyiv-Mohyla Academy, Skovoroda Str., 2, Kiev, 04070, Ukraine; 2 Alcohol and Drug Information Centre (ADIC-Ukraine), Vishnyakovskaya Str., 13-212, Kiev, 02140, Ukraine; 3 National University of Kyiv-Mohyla Academy, Skovoroda Str., 2, Kiev, 04070, Ukraine

## Abstract

**Background:**

Aim: To estimate the impact of smoking restrictions in homes and schools, and tobacco advertising and information on smoking initiation by young people in Ukraine.

**Methods:**

Data of 609 young people aged 15–29 was taken from the national representative survey conducted in June 2005. Outcome measures: The reported age of cigarette initiation was used to characterize the start of smoking experimentation, and the reported age of daily smoking initiation was considered to be a characteristic of established smoking.

Analysis: survival analysis Cox proportional hazard regression models were used.

**Results:**

Age of smoking initiation was reported by 87% of young men and 61% of young women, the beginning of daily smoking by 71% and 33% respectively. Being frequently exposed to second-hand smoke and having no household smoking restrictions was associated with a higher risk of earlier smoking initiation both for men and women. For women, this risk was associated with age, HR = 0.95, (95% CI 0.91–0.98), that is, younger girls were more likely to smoke their first cigarette earlier in their lifetime. Those women had a higher risk of early smoking initiation who reported to receive tobacco-related information from magazines, HR = 1.40 (1.01–1.92), and outdoor tobacco advertising, HR = 1.99 (1.45–2.75). With both men and women, the risk of establishing daily smoking was higher in those with lower levels of tobacco-related knowledge and less household smoking restrictions. For women, the risk was higher in those who live in larger cities HR = 1.77 (1.10–2.86), and who received information about tobacco smoking from colleagues or friends HR = 1.83 (1.13–2.95).

**Conclusion:**

Encouraging people to eliminate their homes of tobacco smoke and tobacco advertising bans can be effective measures in preventing the initiation of smoking among young people. Young female smoking initiation is of special concern in Ukraine, since they are more responsive to tobacco marketing and pro-smoking peer influence.

## Background

Smoking initiation by young people constitutes one of the main challenges for tobacco control. The causes and conditions which contribute to smoking initiation by young people have been well established [1,2], and much research focused on studying the psychosocial determinants of smoking initiation [3-5]. In order to more fully address youth tobacco use, however, we should also consider how well-developed tobacco control policies could positively affect prevention and awareness and how those measures could be instituted on a national and local level.

The effect of such measures has been documented in those countries which have developed tobacco control legislation [6-9]. However, country specificity needs to be taken into account, as not all public health measures effective in developed countries are as effective in the developing and transitional world.

The smoking initiation among young people in Ukraine is disproportionately high: In 1999, 73.6% of 13–15 aged school-children reported having ever smoked cigarettes, which was the second highest rate in 75 sites where GYTS was conducted in 1999–2001 [10].

Along with these conditions, no tobacco-control legislation was enacted in Ukraine until Autumn 2005. As a result, natural quasi-experiments with pre- and post-measurements can not, as yet, be utilized to establish the effectiveness of certain legislative measures. Different workplaces and households practice various approaches to smoking regulation; people living in urban and rural areas can be more or less exposed to tobacco advertising. Because there is no standardized curriculum or widespread scientifically based educational materials, school teachers may provide increased amount of information on tobacco-related harm or may provide none. Based on this natural or non-legislative diversity of factors which can influence smoking behaviour, we aimed to explore potentially effective interventions that could be implemented through national or community health policies. Through earlier studies [11], we recognize that students in Ukraine were less likely to be smokers than those no longer attending school (when age and gender was controlled). We were particularly interested in those factors potentially related to educational institutions which can be utilized in effective tobacco control interventions. This led to the decision to focus on the age group still involved in the process of study, that is to say, those ages most likely to still be attending school which we identified through surveys as up to 29 years old.

We hypothesize that the following factors contribute to the initiation and continued use of tobacco by young people: Exposure to tobacco advertising, receiving little to no information about tobacco's health impact, reporting little or no smoking restrictions in their homes and schools, and having teachers less concerned or less knowledgeable about the dangers of smoking.

## Methods

This study is based on the data gathered within a national survey conducted in Ukraine in June 2005, [12] before any tobacco control legislation was enacted on either the local or national level. The sampled population represents Ukrainian citizens aged 15 and older who permanently reside on Ukrainian territory, are not engaged in military service, and are not imprisoned or residing in medical facilities. A four-stage sampling of respondents was used, which was random in the first three stages, leading to a quota-based selection at the final stage. The response rate of the survey was 85%. The Kyiv International Institute of Sociology performed data collection. The quota-based sampling developed for this study is representative of Ukraine as a whole, and of its six regions. A comparison of demographic categories with data from the 2001 All-Ukrainian Census to evaluate representativeness revealed a discrepancy of less than 5%. Respondents were interviewed face-to-face in their homes. Participants of the survey were asked about their smoking behaviour according to the WHO recommended standardized questions [13], age of smoking their first cigarette, age of establishing daily tobacco use, exposure to second-hand smoke (SHS), their attitude towards smoking and knowledge of tobacco-related issues, exposure to tobacco advertising, contact to smoking regulations and their attitude towards those regulations. Socio-demographic characteristics were collected as well. The questionnaire was approved by the Ethics Committee at the Kyiv International Institute of Sociology. Informed consent forms were signed by parents for underage respondents.

For the goals of this study, a sub-sample of 609 young people aged 15–29 was considered.

### Outcome measures 

The initiation of smoking was explored by the means of two variables. The reported age of the first cigarette smoked was used to characterize the start of smoking experimentation, and the reported age of daily smoking initiation was considered to be a characteristic of established smoking.

### Independent variables

Respondents were questioned about smoking restrictions in their workplace or educational institution. They could choose one of the following options: (1) Smoking is either totally banned or there are no smokers; (2) Smoking is restricted to particular places in the premises; (3) Places allocated for smoking are also used by non-smokers; and (4) There are no restrictions of smoking at all.

Respondents were asked to specify if there were any restrictions of smoking in their households. The response options were as follows: (1) There are no smokers in the household; (2) Family members and guests smoke only outdoors; (3) Smoking takes place on the stairs, balcony etc.; (4) Smoking is limited to certain parts of the house or flat; (5) Smoking is not restricted. The first two options together reflected adequate protection of family members against tobacco smoke and are referred further as 'strict household smoking restrictions'.

To explore advertising exposure, respondents were asked a multiple-choice question: Where do you notice advertisements for tobacco products? (1) Television; (2) Radio; (3) Outdoor ads/posters; (4) Newspapers/magazines; (5) Promotional items (pens, lighters, etc.); (6) Bars/restaurants/shops. Those who reported a specific option were considered exposed to that kind of tobacco advertising.

To measure the level of tobacco-related knowledge, respondents were asked to choose which of the following statements were correct: (1) People have the right to work in a smoke-free environment; (2) Light and ultra-light cigarettes are safer than regular ones; (3) Tobacco contains substances that cause addiction; (4) Smoking causes only lung cancer and respiratory diseases; (5) Passive smoking can lead to illness and even death; (6) Smokers suffer from Parkinson's disease more often that non-smokers; (7) Smokers run an increased risk of developing impotence; (8) Smokers can easily give up smoking, as long as they want to; (9) Mothers who smoke give birth to babies that are smaller than average and suffer from a variety of diseases; (10) It is safe to smoke for a few years if you give up eventually; (11) Tobacco smoke causes heart and circulatory diseases.

The integrated knowledge index was calculated per the number of correct answers to these 11 statements related to knowledge and beliefs. It varied from 1 to 11 for all respondents. Respondents were then classified into three groups: 'low knowledge' – with 1 to 5 correct answers, 'medium knowledge' – with 6 correct answers, 'high knowledge' – 7 to 11 correct answers.

Sources of tobacco-related knowledge were studied by means of the following multiple-choice question: From what sources do you normally receive information about tobacco products, their impact on human health and political efforts to regulate this issue? (1) Newspapers; (2) Magazines; (3) Radio; (4) Television; (5) Internet; (6) Lessons at school or other educational institution; (7) Medical workers; (8) Contact with family members; (9) Contact with friends or colleagues; (10) I don't receive this kind of information.

### Analysis

As both outcome measures are time-to-event data, a survival analysis was used to estimate risks. For those who reported the event (first cigarette or daily smoking), the time of observation lasted from birth to the reported age of smoking initiation. For the others (who were censored cases) the time of observation was from their birth to the time of interview.

The analysis of the risk to establish daily smoking was performed only among those who reported their age of smoking initiation. The outcome measure in this case was not the risk of being a daily smoker but the risk for advancing from experimenting to daily smoking.

To estimate risks associated with independent variables, Cox Proportional Hazard Regression Models were used. Socio-demographic characteristics and tobacco control-related variables were analysed in bivariate analysis, then socio-demographic characteristics associated with outcome measures were considered as potential confounders and were controlled for in multivariate Cox Proportional Hazard Regression Models. To estimate the mean age of smoking initiation in particular groups, the Kaplan-Meier analysis was used.

All the associations were analysed separately for men and women. This was due to the fact that gender was found to be the strongest correlate of both outcome measures. Additionally we have earlier evidence of different correlates for female and male smoking in Ukraine. [14]

Survival analysis was performed using the SPSS 10.05 standard version for Windows. Only those hazard ratios which significantly differ from 1.00 are reported.

## Results

The details of the samples are presented in Table 1. On average, women in the sample were higher educated than men, though the proportion of students among them was the same. About 60% of study participants were inhabitants of cities. More than 31% of men and 23% of women reported their income to be lower than middle.

**Table 1:** Study group characteristics by gender

	Men				Women			
	N	% or Mean	95%Confidence Interval		N	% or Mean	95% Confidence Interval	

Total number	316		Lower bound	Upper bound	293		Lower bound	Upper bound

Reported the age of first cigarette	274	86,70%	82,96%	90,44%	179	61,10%	55,52%	66,68%
Reported the age of established daily smoking	225	71,20%	66,21%	76,19%	97	33,10%	27,71%	38,49%
Mean age at interview		22,36	21,90	22,82		22,69	22,18	23,19
Mean age of first cigarette		13,53	13,09	13,97		15,96	15,44	16,47
Mean age of established daily smoking		16,68	16,33	17,02		17,82	17,18	18,47
Education								
Primary	47	14,90%	10,97%	18,83%	34	11,60%	7,93%	15,27%
Secondary	138	43,70%	38,23%	49,17%	119	40,80%	35,17%	46,43%
College	72	22,80%	18,17%	27,43%	74	25,30%	20,32%	30,28%
University	59	18,70%	14,40%	23,00%	65	22,30%	17,53%	27,07%
Students	106	33,50%	28,30%	38,70%	99	33,80%	28,38%	39,22%
Type of settlement								
city with >1 mln inhabitants	96	30,40%	25,33%	35,47%	93	31,70%	26,37%	37,03%
City	93	29,40%	24,38%	34,42%	85	29,00%	23,80%	34,20%
Town	59	18,70%	14,40%	23,00%	56	19,10%	14,60%	23,60%
Village	68	21,50%	16,97%	26,03%	59	20,10%	15,51%	24,69%
Self reported income lower than middle	91	31,60%	26,47%	36,73%	64	23,60%	18,74%	28,46%
Self reported mobile telephone for personal use	190	60,30%	54,91%	65,69%	169	57,90%	52,25%	63,55%
Perceived SHS exposure daily or more frequently	213	68,90%	63,80%	74,00%	189	64,50%	59,02%	69,98%
Smoking restrictions at workplace or educational institution								
(1) smoking is either totally banned or there are no smokers	35	12,50%	8,85%	16,15%	48	21,70%	16,98%	26,42%
(2) smoking is restricted to particular places in the premises	94	33,50%	28,30%	38,70%	80	36,20%	30,70%	41,70%
(3) places allocated for smoking are also used by non-smokers	60	21,40%	16,88%	25,92%	48	21,70%	16,98%	26,42%
(4) there are no restrictions on smoking at all	92	32,70%	27,53%	37,87%	45	20,40%	15,79%	25,01%
Restrictions of smoking in the households								
(1) There are no smokers in the household	59	18,80%	14,49%	23,11%	77	26,30%	21,26%	31,34%
(2) Family members and guests smoke only outdoors	105	33,40%	28,20%	38,60%	87	29,70%	24,47%	34,93%
(3) Smoking takes place on the stairs, balcony etc.	87	27,70%	22,77%	32,63%	65	22,20%	17,44%	26,96%
(4) Smoking is limited to certain parts of the house or flat	46	14,60%	10,71%	18,49%	47	16,00%	11,80%	20,20%
(5) Smoking is not restricted	17	5,40%	2,91%	7,89%	17	5,80%	3,12%	8,48%
Summary of tobacco-related knowledge								
1–5 correct answers	122	38,60%	33,23%	43,97%	80	27,30%	22,20%	32,40%
6 correct answers	87	27,50%	22,58%	32,42%	97	33,10%	27,71%	38,49%
7–11 correct answers	107	33,90%	28,68%	39,12%	116	39,60%	34,00%	45,20%
Reported contact with tobacco advertising								
TV	78	24,70%	19,94%	29,46%	98	33,40%	28,00%	38,80%
Radio	6	1,90%	0,39%	3,41%	10	3,40%	1,32%	5,48%
Outdoor advertising	218	69,00%	63,90%	74,10%	177	60,40%	54,80%	66,00%
Newspapers and magazines	31	9,80%	6,52%	13,08%	39	13,30%	9,41%	17,19%
Advertising goods	42	13,30%	9,56%	17,04%	27	9,20%	5,89%	12,51%
Bars/restaurants/shops	64	20,30%	15,87%	24,73%	46	15,70%	11,53%	19,87%
Sources of information about tobacco products, their impact on human health and political efforts to regulate this issue								
(1) Newspapers	85	26,90%	22,01%	31,79%	84	28,70%	23,52%	33,88%
(2) Magazines	62	19,60%	15,22%	23,98%	77	26,30%	21,26%	31,34%
(3) Radio	27	8,50%	5,43%	11,57%	28	9,60%	6,23%	12,97%
(4) Television	162	51,30%	45,79%	56,81%	140	47,80%	42,08%	53,52%
(5) Internet	8	2,50%	0,78%	4,22%	7	2,40%	0,65%	4,15%
(6) Lessons at school or other educational institution	38	12,00%	8,42%	15,58%	38	13,00%	9,15%	16,85%
(7) Medical workers	25	7,90%	4,93%	10,87%	28	9,60%	6,23%	12,97%
(8) Contact with family members	42	13,30%	9,56%	17,04%	29	9,90%	6,48%	13,32%
(9) Contact with friends or colleagues	55	17,40%	13,22%	21,58%	49	16,70%	12,43%	20,97%
(10) I don't receive this kind of information	59	18,70%	14,40%	23,00%	53	18,10%	13,69%	22,51%

The age of tobacco initiation was reported by 87% of young men and 61% of young women, the beginning of daily smoking by 71% and 33% respectively. The mean reported age of smoking initiation was 13.5 years for men and about 16.0 for women. Time between the first cigarette and the beginning of daily smoking was longer for men (about three years) than for women (less than two years).

Between 60 and 70% of young people in Ukraine reported being exposed to tobacco smoke on a daily basis, 54% of men and 42% of women are exposed in places of work or education, 48% of men and 44% are exposed in their homes. Compared to other types of advertising, outdoor tobacco advertising was most frequently seen by young people, as reported by 69% of men and 60% of women.

### Correlates of smoking initiation

Variables associated with the age of smoking initiation are shown in Table 2. Socio-demographic characteristics found to be associated with the age of the first cigarette were different for men and women.

**Table 2:** Multivariate survival analysis of age of first cigarette smoked (adjusted Cox proportional hazard ratios – controlled for socio-demographic characteristics associated with smoking initiation)

	Men*	Women**
Age		0,95 (0,91–0,98)
Living in a city versus a village		1,77 (1,10–2,86)
Number of people in the household		0,82 (0,73–0,93)
Income lower than middle versus middle or higher	1,27 (0,98–1,66)	
Household smoking restrictions (Smoke outdoors versus no restrictions)	0,78 (0,61–0,99)	0,39 (0,28–0,53)
Exposure to tobacco smoke rare versus frequent	0,84 (0,73–0,97)	0,79 (0,66–0,94)
Outdoor advertising reported		1,62 (1,16–2,27)
Tobacco-related knowledge	0,89 (0,82–0,97)	
Low knowledge versus high knowledge	1,60 (1,14–2,24)	
Receiving information about tobacco from magazines		1,43 (1,03–1,97)

* HR adjusted for income level

** HR adjusted for age, type of settlement and family size

Men were more likely to smoke their first cigarette earlier if they reported low income, and if they had low knowledge of tobacco-related issues. Being frequently exposed to SHS and having no household smoking restrictions was also associated with higher risk of earlier smoking initiation.

For women, risk was associated with age, that is, those girls who were born later were more likely to smoke their first cigarette earlier in lifetime than those born earlier: for women born in 1975–80 mean age of smoking initiation was 22, for those born in 1981–85 the mean age was 19, and for those born in 1986–90 smoking started on average at the age 16. Risk was higher in those women who live in larger cities and in smaller families. Like men, women were more likely to start smoking earlier if they were more frequently exposed to SHS, reported no strict smoking regulations in their homes, and witnessed tobacco advertising on billboards. Those women who reported obtaining tobacco-related information from magazines had a higher risk of early smoking initiation.

### Correlates of establishing daily smoking

Multivariate survival analysis is presented in Table 3. Both for men and women, the risk of establishing daily smoking was inversely associated with age, level of tobacco-related knowledge and household smoking restrictions. For men, the risk of becoming a daily smoker was higher in those with lower education and more exposure to SHS. Several variables related to income level, which were primarily associated with the risk of advancing to daily smoking, were excluded from the model after controlling for age and education. For women, the risk was higher in those who live in larger cities and get information about tobacco smoking from colleagues or friends.

**Table 3:** Multivariate survival analysis of the reported age of established daily smoking (adjusted Cox proportional hazard ratios and their 95% confidence intervals for socio-demographic and tobacco control-related characteristics)

	Men	Women
	N = 279	N = 182

Age	0,95 (0,91–0,98)	0,92 (0,87–0,97)
Education (primary versus university)	2,60 (1,61–4,20)	
Smaller versus larger settlements		0,77 (0,63–0,95)
Exposure to tobacco smoke rare versus frequent	0,67 (0,49–0,92)	
Household smoking restrictions (Smoke outdoors versus no restrictions)	0,64 (0,49–0,84)	0,60 (0,39–0,93)
Tobacco-related knowledge	0,76 (0,57–1,02)	0,83 (0,72–0,95)
Receive information on tobacco smoking from colleagues and friends		1,83 (1,13–2,95)

## Discussion

Our study has shown that adolescent smoking is a growing problem in Ukraine. While earlier reports found that young adults have higher smoking prevalence than people in older age groups[14], this study has additionally shown that younger girls and women in Ukraine now have a higher risk of smoking their first cigarette earlier than had those born earlier: the mean age of smoking initiation in women decreases by 3 years per every 5 year time span. Both men and women have now greater risk of proceeding towards daily smoking than those from several years ago. This makes the need of tobacco control measures more urgent.

Different correlates of smoking initiation and transition to daily smoking in men and women give evidence that a smoking epidemic in men and women in Ukraine are in different stages of development. In men the risk of smoking initiation is higher in those having lower income, less educated, and less informed about dangers of smoking. In Ukrainian men smoking has the same correlates as in developed countries with a long history of tobacco control measures. This result is in agreement with other observations showing that smoking prevalence is lower among men with a university education than among those with primary or secondary education [14].

Conversely, the risk of smoking initiation is higher in those women in Ukraine who live in large cities and have smaller families. Thus, the smoking epidemic among women is developing in parallel with an urbanization and emancipation process.

Those women more likely initiate smoking who report seeing outdoor tobacco advertising, and report receiving tobacco-related information from magazines. Additionally, women receiving information on tobacco smoking from colleagues and friends are more likely to develop a daily smoking habit. Which comes first and whether the association is causal is not possible to define in a cross-sectional study.

### Study limitations

The findings of this study are subject to several limitations. The most important limitation is typical for all cross-sectional studies, which mainly allow to generate hypotheses but not to test them. Causal inferences are beyond this study design, and we only can hypothesize whether women start smoking because they see outdoor advertising and direct or hidden ads in the magazines, or they notice these ads because they are already experimenting with smoking.

Another limitation is that some respondents mentioned only the age of daily smoking initiation and not the age of their first cigarette. We tried to overcome this by utilizing only those who mentioned at least one age in the second analysis. However, the group analysed for the correlates of establishing daily smoking was not precisely the subgroup of those who reported the age of their first cigarette. Several distortions could be possible due to recall bias though it is difficult to fully estimate its extent.

Associations found in this study can be further explored through the prospective study design. Further elaborations may include the analysis of smoking initiation processes in narrower age groups through surveys of larger groups of young people. More specific developments of the present findings related to the correlates of female smoking initiation can be expressed in qualitative studies among young women aimed at revealing peculiarities of tobacco marketing which are more likely to hook this age group, with elaboration of counter-marketing strategies.

### Impact of household smoke-free regulation

Those correlates which are common for both men and women and for both smoking initiation and daily smoking establishment are related to smoking restrictions and SHS exposure. Those reporting their homes as being smoke-free and themselves as being less exposed to SHS were less likely to initiate smoking. In this study, we found correlates only with household smoking regulations, while our earlier attempts [11] to explore correlates of smoking status in young people have shown associations with workplace smoking regulations.

A factor in this research may be that young adults are just entering the workforce and thus policies in these institutions could not influence smoking behaviour within the previous several years. Household conditions can be, generally speaking, more stable. This may be why only household correlates, not workplace smoking regulations were found to influence tobacco initiation and use amongst this age group.

Earlier research has shown that parental and other household factors can predict adolescent smoking initiation[2]. Farkas et al. have shown that adolescents who lived in smoke-free households were 74% (95% confidence interval [CI], 62%–88%) as likely to be smokers as adolescents who lived in households with no smoking restrictions [7]. The mechanisms by which smoke-free homes can influence the smoking behaviour of their family members are explored by Gilpin et al[15]. Both direct and indirect mechanisms can be involved, for example: among adolescents, a household smoking ban was associated with a lower perceived prevalence of adult smoking in their communities and more negative attitudes regarding the social acceptability of smoking. These two factors would therefore affect the likelihood of smoking initiation[16].

Our results show that smoking initiation is associated with household smoking regulations to a larger extent among young women than men. Male smoking is more socially accepted in Ukraine and is therefore more influenced by social environments, and in particular, job conditions. Comparatively concealed female smoking is more affected by home conditions.

### Impact of tobacco advertising

Our findings show that reported exposure to tobacco advertising (when respondents report to have noticed specified types of tobacco advertising) is associated with a higher probability of smoking initiation among men and women. This is in line with research indicating that pro-tobacco media and advertising increases susceptibility to smoking over time,[17] that tobacco advertising and promotion increases the likelihood that adolescents will start to smoke [18-20], and that a causal relationship exists between tobacco promotion and smoking initiation among adolescents[21]. Our research gives additional arguments to those politicians in Ukraine who propose to ban tobacco advertising in the country, or at the very least, the outdoor advertising.

We found an association only with the outdoor advertising, which may be explained through the high visibility of outdoor advertising compared with other kinds like advertising goods, point of sale advertising etc. It appears that there is a greater heterogeneity in exposure to outdoor tobacco advertising, as it is seen less in the rural areas than in urban ones. Yet, we noted that when the type of settlement was controlled there was still a significant association between outdoor advertising and smoking initiation. We found that other types of advertising like advertising goods and point of sale advertising are more evenly seen throughout the country, and possibly more often viewed where outdoor advertising is less reported.

It is noteworthy that we did not find a dose-and-effect relationship between advertising exposure and the risk of smoking initiation and progression. We only surveyed the extent of contacts with all the types of advertising together, not specifically outdoor advertising.

Our finding that for young women there is a stronger association between the tobacco advertising and smoking initiation than among men is probably explained by the tobacco industry promotional campaigns aimed at young women, [22,23] and the fact that the tobacco industry may target women in a manner that differs from its targeting of men[24].

### Impact of tobacco-related knowledge

We found that among adolescents and young adults, better knowledge of tobacco-related harm was negatively associated with daily smoking initiation, while impact of tobacco-related knowledge on the age of smoking initiation is negligible. The present study results are in line with the international evidence that the mass-media interventions can be effective in preventing the uptake of smoking in young people, [8] and supports the need for wide-range media campaigns, which can result both in smoking cessation and prevention of smoking initiation.

The questions regarding sources of tobacco-related information, which was explained as information about tobacco related harm and tobacco control measures, did not reveal any protective influences. The hypothesis of the protective role of school-based information was not supported. Both sources which showed significant associations, if reported, were associated with a higher risk of smoking initiation. While this could be expected with information coming from friends and colleagues who in this case were most likely smokers, the association between a higher risk of smoking initiation and information coming from magazines needs to be further discussed. We suspect that girls noticed direct and indirect tobacco advertising found in female magazines and responded about this rather than health-related information. The revealed association shows the necessity to consider a tobacco advertising ban in printed media as one of the tobacco control priorities in Ukraine.

## Conclusion

Among Ukrainian young people aged 15–29 smoking initiation is very high: 87% of men and 61% of women. Among the surveyed young people 71% of men and 33% of women proceeded towards daily smoking.

Smoking regulation within households which only allowed smoking outdoors was associated with a lower smoking incidence among young people. Encouraging people to eliminate tobacco smoke exposure in their homes can be an effective measure in preventing the initiation of smoking among young people. Being exposed to outdoor tobacco advertising was associated with an increased chance of early smoking initiation and continuation, especially among females. A tobacco advertising ban can be an effective tool in reducing smoking initiation. The implementation of comprehensive advertising bans can be started with the ban of outdoor advertising.

The study reveals that young female smoking initiation is of special concern in Ukraine: the young women smoking initiation age is now much earlier than 10–15 years ago; they are more responsive to tobacco marketing and pro-smoking peer influence. Additional studies are needed to develop gender-specific smoking prevention tools to stop smoking prevalence increase among Ukrainian young women.

## Competing interests

The author(s) declare that they have no competing interests.

## Authors' contributions

TA coordinated the preparation of the survey questionnaire, analysed the data and drafted the article. KK participated in the questionnaire preparation, discussion of the study idea and design, critically revised the manuscript. DS participated in the discussion of the study idea and design, critically revised the manuscript. All authors read and approved the final manuscript.

## Pre-publication history

The pre-publication history for this paper can be accessed here:


